# Periprocedural heparin bridging in patients receiving oral anticoagulation: a systematic review and meta-analysis

**DOI:** 10.1186/s12872-017-0719-7

**Published:** 2017-12-13

**Authors:** Jing Wen Yong, Li Xia Yang, Bright Eric Ohene, Yu Jie Zhou, Zhi Jian Wang

**Affiliations:** 1 Beijing Institute of Heart Lung and Blood Vessel Disease, The Key Laboratory of Remodeling-related Cardiovascular Disease, Ministry of Education, Anzhen Hospital, Capital Medical University, Beijing, China; 2 Department of Cardiology, Beijing Anzhen Hospital, Capital Medical University, Anzhen Avenue #2, Chaoyang district, Beijing, 100029 China

**Keywords:** Anticoagulation, Bridging, Heparin, Meta-analysis

## Abstract

**Background:**

Periprocedural heparin bridging therapy aims to reduce the risk of thromboembolic events in patients requiring an interruption in their anticoagulation therapy for the purpose of an elective procedure. The efficacy and safety of heparin bridging therapy has not been well established.

**Objectives:**

To compare through meta-analysis the effects of heparin bridging therapy on the risk of major bleeding and thromboembolic events of clinical significance among patients taking oral anticoagulants.

**Methods:**

We searched PubMed, EMBASE and the Cochrane library from January 2005 to July 2016. Studies were included if they reported clinical outcomes of patients receiving heparin bridging therapy during interruption of oral anticoagulant for operations. Data were pooled using random-effects modeling.

**Results:**

A total of 25 studies, including 6 randomized controlled trials and 19 observational studies, were finally included in this analysis. Among all the 35,944 patients, 10,313 patients were assigned as heparin bridging group, and the other 25,631 patients were non-heparin bridging group. Overall, compared with patients without bridging therapy, heparin bridging therapy increased the risk of major bleeding (OR = 3.23, 95%CI: 2.06–5.05), minor bleeding (OR = 1.52, 95%CI: 1.06–2.18) and overall bleeding (OR = 2.83, 95%CI: 1.86–4.30).While there was no significant difference in thromboembolic events (OR = 0.99,95%CI: 0.49–2.00), stroke or transient ischemic attack(OR = 1.45, 95%CI: 0.93–2.26,) or all-cause mortality (OR = 0.71, 95%CI: 0.31–1.65).

**Conclusions:**

Heparin-bridging therapy increased the risk of major and minor bleeding without decreasing the risk of thromboembolic events and all cause death compared to non-heparin bridging.

Long-term anticoagulation is used in relatively large population of patients to treat and prevent thromboembolic events. However, each year, at least 10% of patients on oral anticoagulants require treatment interruption for surgery or an invasive procedure associated with a bleeding risk [1]. Perioperative heparin bridging (HB) is common in clinical practice,but its safety and efficacy are not yet established. A large randomized pacemaker or defibrillator trial reported significantly lower rate of hematoma in uninterrupted, non-HB oral anticoagulation therapy compared with the bridging therapy (3.5% vs. 16.0%) [2]. A meta-analysis found that vitamin K antagonist (VKA)-treated patients who received bridging therapy were at increased risk of bleeding events but similar risk of thromboembolic events compared with non-bridged patients [3]. However,it was lack of randomized controlled trial to justify or prove otherwise the results.

In recent years, increasing number of studies continue to demonstrate the practical advantages (safer) of continuous oral anticoagulation approach over perioperative HB. Notwithstanding, most of these studies are underpowered to drawing firm conclusion owing to small nature of their sample size. Presently, the position of the practical guidelines on the clinical application of Bridging therapy is not neither firm nor consistent possibly due to lack of high-quality evidence [4]. Given the uncertainties associated with optimal periprocedural anticoagulant management and the use of bridging therapy, we performed a systematic review and meta-analysis of current studies to evaluate the safety and efficacy of periprocedural bridging therapy among patients taking oral anticoagulants.

## Methods

### Search strategy

The guideline of the MOOSE (Meta-analysis of Observational Studies in Epidemiology) was meticulously followed for the conduction of the current systematic review and meta-analysis [5].We searched PubMed, EMBASE and the Cochrane Central Register of Controlled Trials for current literature. Detailed search strategies are demonstrated in the eMethods. The last search was performed on July 1, 2016. Reference lists from these identified reports and reviews were manually screened to identify additional relevant studies. To minimize the heterogeneity due to the rapidly advancing diagnostic techniques and treatment strategies, we only included studies published from January 1, 2005. The search was limited to studies in human adults published in peer-reviewed journals. Studies in abstract form without a published manuscript were excluded. We only included articles written or published in English.

### Study selection

Two investigators (J.W.Y. and L.X.Y.) re-screened the titles and abstracts of all retrieved literature independently. Then full-text reports considered relevant were assessed for eligibility for inclusion. Disagreement was resolved by discussion and consulting a third investigator (Z.J.W.). Studies were considered eligible for this review if: 1) compared between heparin bridging therapy and control group (including interruption of OAC or continuation of OAC or without OAC) among patients taking oral anticoagulants and undergoing an elective operation or other elective invasive procedure; 2)reporting clinical outcomes including bleeding or thromboembolic events; and 3)involved >100 patients. We defined heparin bridging therapy as interrupting the OAC before an invasive procedure and changing to heparin or low molecular weight heparin.

### Data extraction

Two investigators(J.W. Y. and L.X.Y.) extracted the data from the full reports of the included studies independently and in duplicate. The data included first author, journal, publication year, study population, baseline clinical characteristics and outcomes of patients according to whether or not heparin bridging therapy was employed. Authors of the papers were individually contacted by email when the data were unclear or to obtain additional data. Discrepancies between the two investigators were resolved by consensus involving discussion with the third commentator(Z.J.W.).

The primary safety and efficacy end points were major bleeding and stroke or stroke or transient ischemic attack. Secondary end points included overall bleeding, thromboembolic events, venous thromboembolic events, MI and all-cause mortality. We used the reported definitions of major bleeding provided in the original studies, which included bleeding at critical sites such as intracranial, retroperitoneal, intraocular bleeding causing blindness, or joint hemorrhage; clinically overt bleeding requiring admission; transfusion of ≥2 units of packed red blood cells/whole blood; surgery or angiographic procedures; or fatal bleeding that caused death. The venous thromboembolic events include deep vein thrombosis and pulmonary embolism [6].

### Statistical analysis

Individual study odds ratios (ORs) and 95% CIs were calculated for each article. The I^2^ test was used to assess the heterogeneity between the studies, where *P* < 0.1 is considered heterogeneity. Given the potential high degree of heterogeneity across individual studies, we performed subgroup analyses according to study design, year of study, type of disease, study area, type of anticoagulant, patient number and type of heparin. Meta-regression analyses were subsequently performed. The pre-defined covariates included study sample size, follow-up time, mean age, proportion of men, diabetes mellitus and hypertension. Publication bias was assessed using Egger’s linear regression test, visual inspection of funnel plots, and Begg’s test. The trim-and-fill method was used to adjust for publication bias. Analyses were performed by using Stata 11.0 (Stata Corp, College Station, Tex). Statistical level of significance for the summary estimates was a two-tailed *p* value <0.05.

## Results

**Fig. 1:**
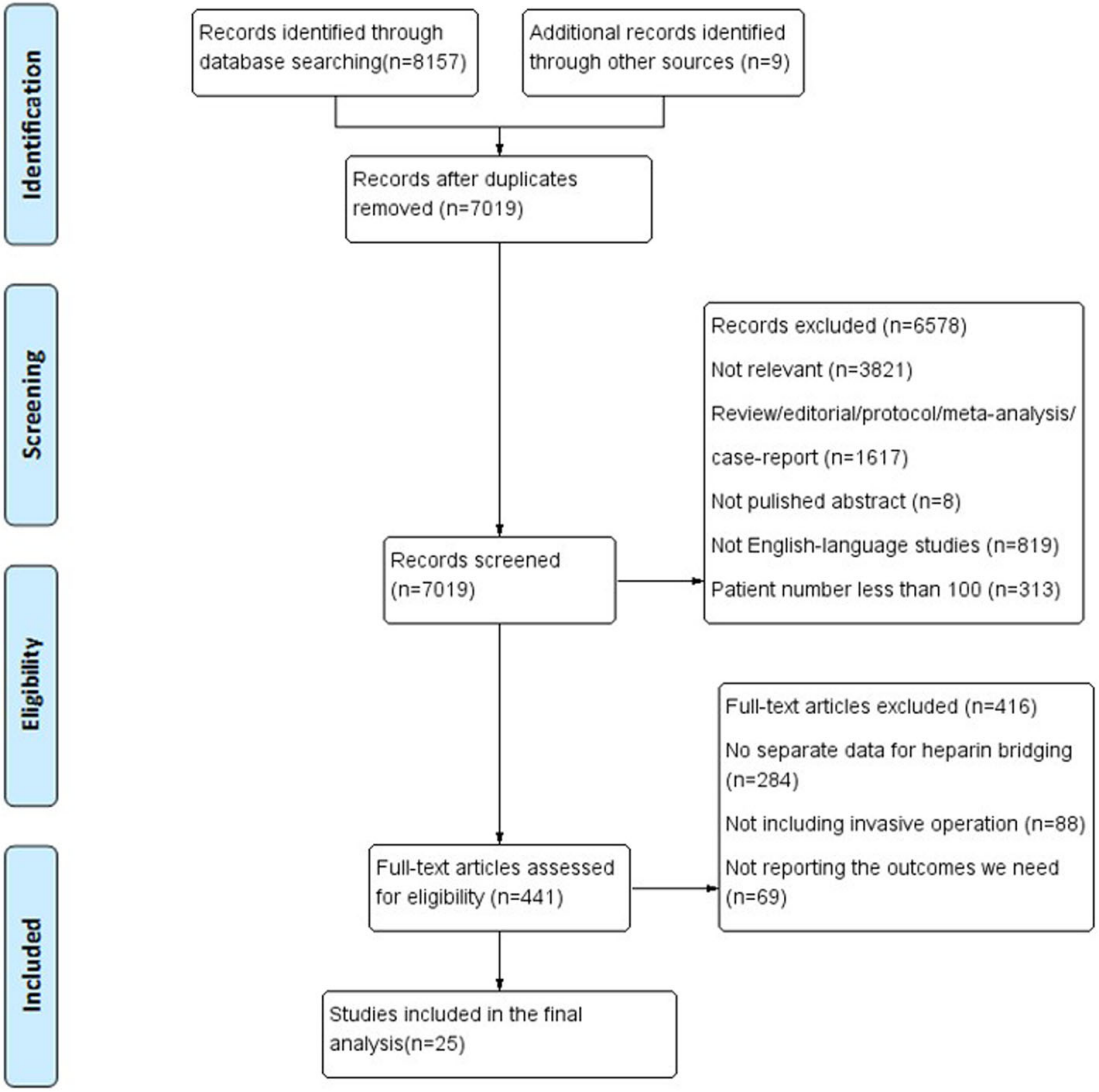
Flow chart of study selection

Our literature search yielded 8166 relevant articles after duplication removed. After exclusion, we finally identified 25 studies, including 19 observational studies and 6 randomized controlled trials. Overall, we included a total of 35,944 patients (average age 70.9 ± 8.1 years) with average CHADS-2 score ranging between 1.80 and 4.03 (mean 2.5 ± 1.1), of whom 10,313 patients received heparin-bridging treatment (HB), and the other 25,631 were assigned as non-heparin bridging group (NHB). Warfarin was used as the coagulation therapy in 19 studies, and the other 7 studies used new oral anticoagulants or mixed. The follow-up period ranged from 7 days to 12 months (Fig. 1).

### Bleeding events

**Fig. 2:**
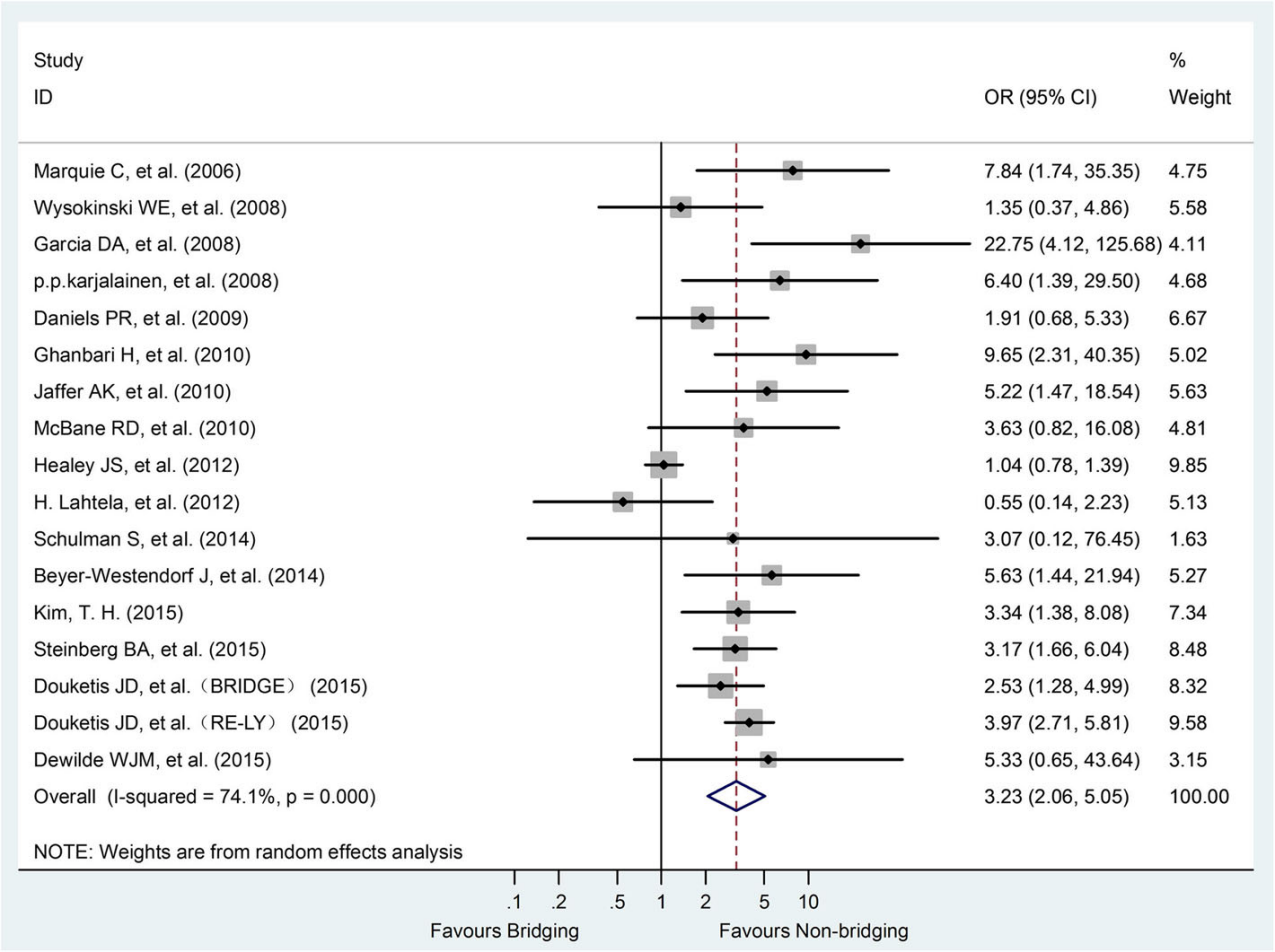
Forest plot of major bleeding for heparin-bridging and non-heparin bridging regiments

Seventeen studies, including 286 patients receiving HB and 267 patients NHB, reported incidence of major bleeding. The median follow-up time was 3.3 months. Compared with NHB, HB was associated with a significantly increased risk of major bleeding (OR = 3.23, 95%CI: 2.06–5.05, *P* < 0.001).There was a high-level heterogeneity across the eligible studies (I^2^ = 74.1%, P < 0.001) (Fig. 2).

**Fig. 3:**
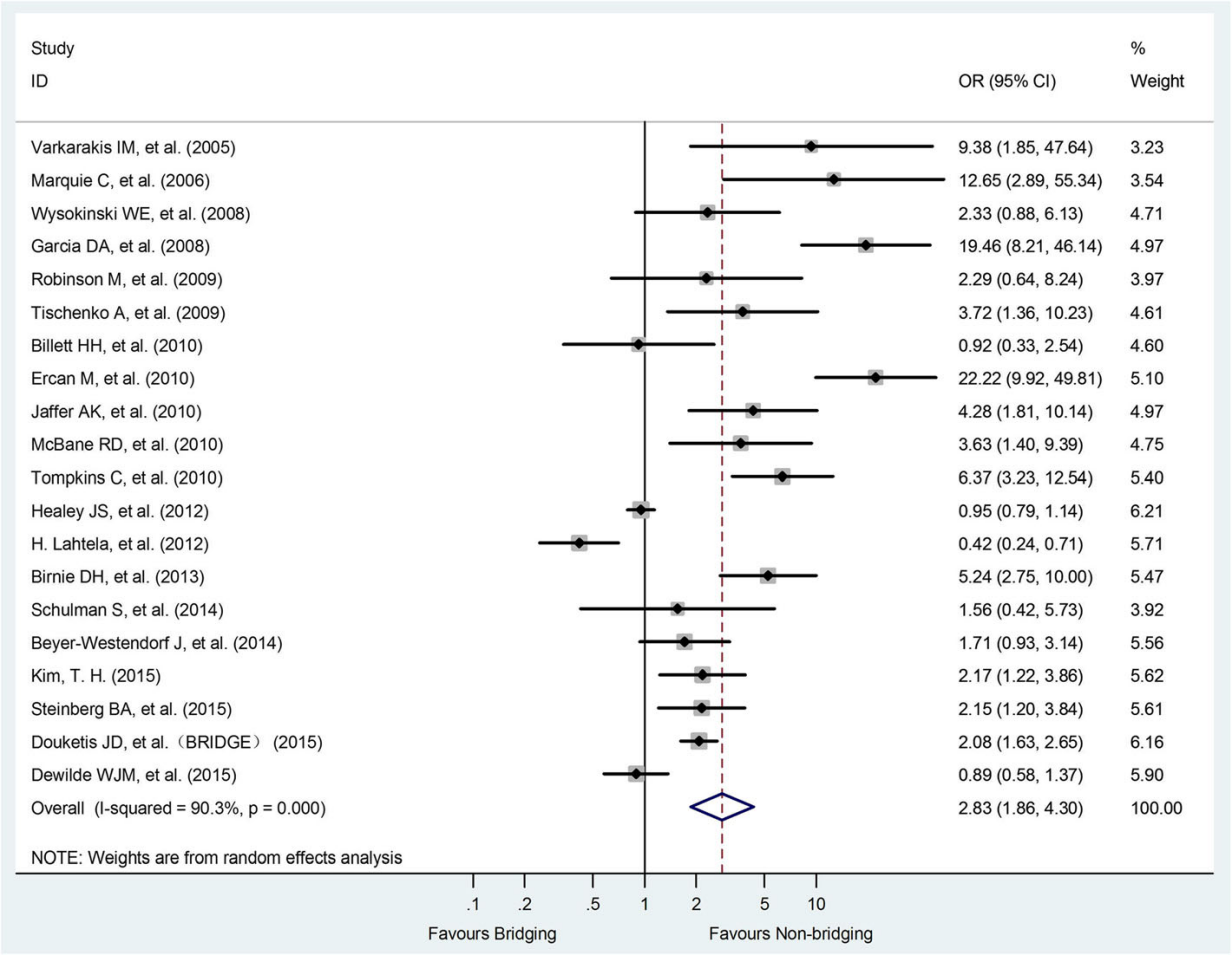
Forest plot of overall bleeding for heparin-bridging and non-heparin bridging regiments

In addition, the HB anticoagulation regimen also increased the risk of minor bleeding events (OR = 1.52, 95% CI: 1.06–2.18, P < 0.001) and overall bleeding risk (OR = 2.83, 95% CI: 1.86–4.30, P < 0.001) (Fig. 3).

### Thromboembolic events

**Fig. 4:**
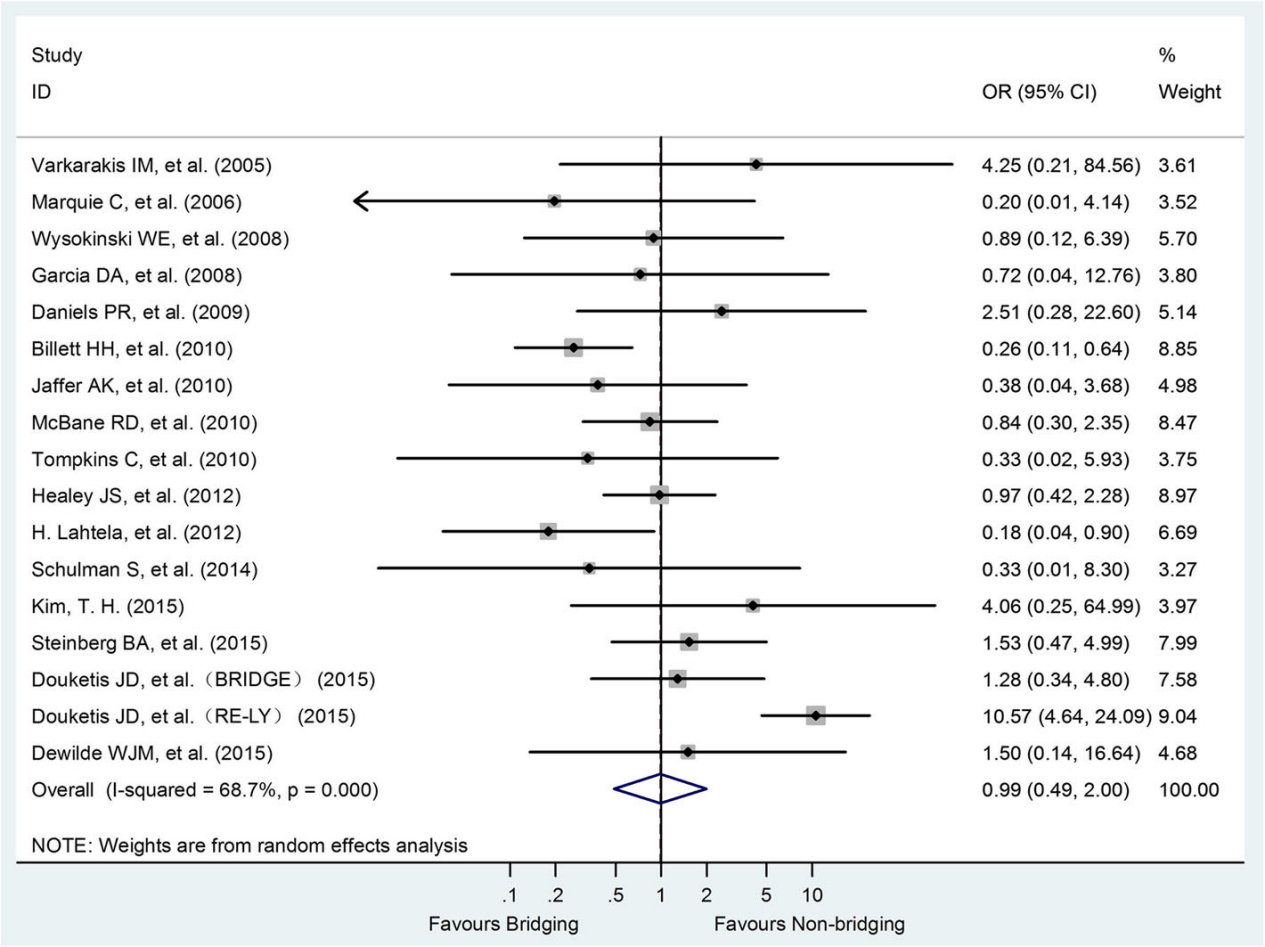
Forest plot of thromboembolism for heparin-bridging and non-heparin bridging regiments

Seventeen studies with a total of 448 patients, including 64 in HB group and 384 in NHB group, provided data on thromboembolic events. Meta-analysis showed that perioperative HB did not reduce thromboembolic events compared with NHB (OR = 0.99, 95% CI: 0.49–2.00, *P* = 0.973).There was also a significant heterogeneity between studies (I^2^ = 68.7%, *P* < 0.001) (Fig. 4).

### Other outcomes

**Fig. 5:**
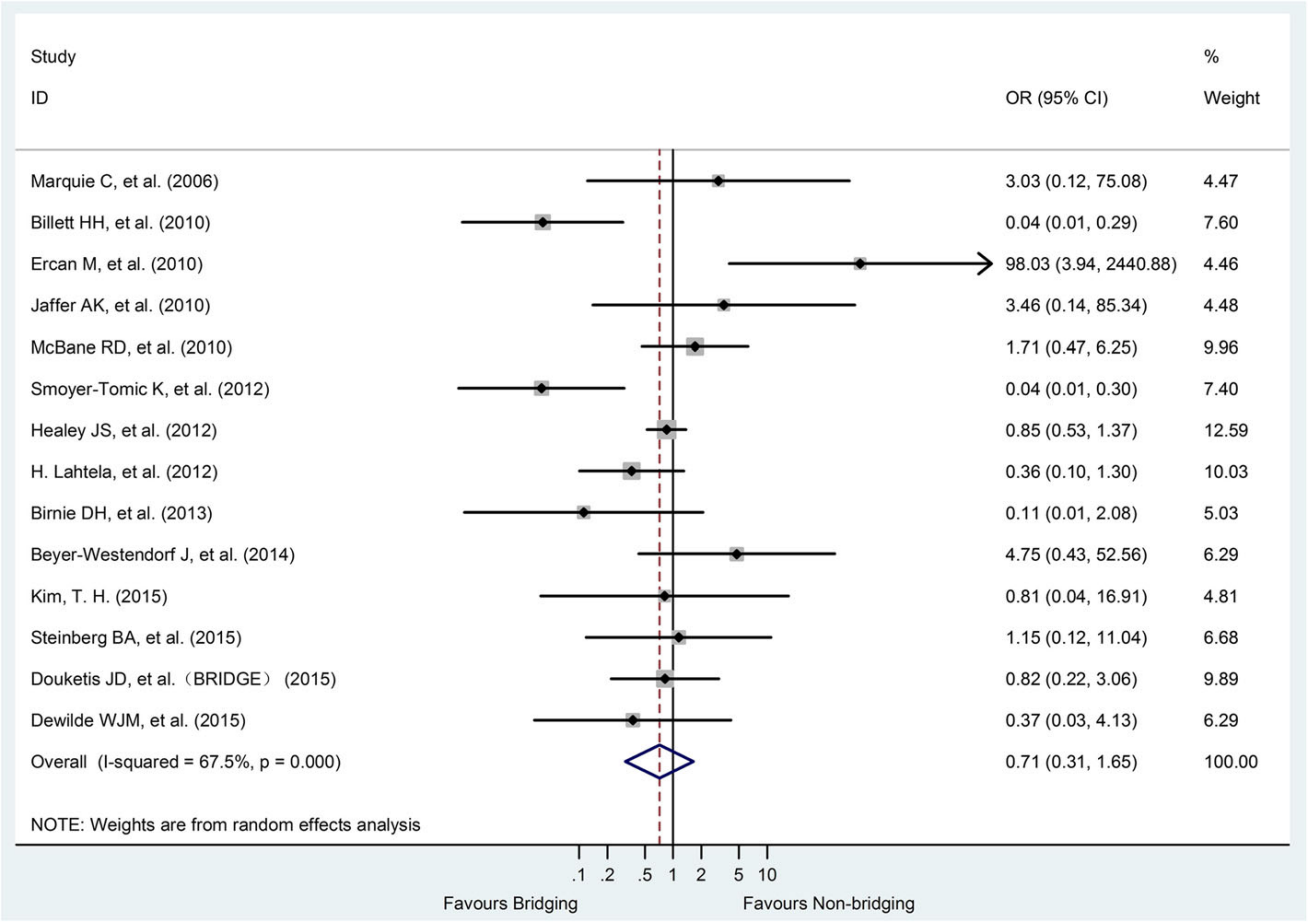
Forest plot of all cause death for heparin-bridging and non-heparin bridging regiments

Fourteen studies provided data on all-cause deaths, with a total of 533 patients, including 52 HBs and 481 NHB. There was significant heterogeneity between studies (I2 = 67.5%, P < 0.001), so the data were pooled using a random-effect model. Meta-analysis showed that perioperative HB did not reduce all-cause mortality (OR = 0.71, 95% CI: 0.31–1.65, *P* = 0.431) compared with NHB (Fig. 5).

In addition, we found similar risk of myocardial infarction (OR = 1.15, 95% CI: 0.70–1.87, *P* = 0.588) and stroke or TIA(OR = 1.45,95% CI:0.93–2.26, *P* = 0.099) between the two treatment strategies.

### Meta regression and subgroup analysis

Meta-regression analysis showed that none of the variables including study sample size, follow-up time, or publication year modified the effect of bridging on major bleeding or thromboembolism. Similarly, according to the subgroup analysis of the study type, publication year, disease, area, type of oral anticoagulant, anticoagulation strategy of the control group, and sample size, we did not find significant differences in the effect of bridging on major bleeding or thromboembolism between the subgroups (Table 1).

**Table 1:** Subgroup analysis

Classification criteria	Subgroups	No. of Studies	*P* value	OR (95% CI)	I-squared (%)	P value for within-group heterogeneity	P value for between-group heterogeneity
Major bleeding							
Year of study	Before2010	8	<0.001	4.63(2.48,8.64)	38.9	0.120	0.136
	After 2010	9	0.002	2.47(1.39,4.38)	80.9	<0.001	
Type of disease	Atrial fibrillation(AF)	6	<0.001	2.66(1.73,4.08)	47.9	0.087	0.244
	Others	11	<0.001	4.50(2.12,9.51)	75.4	<0.001	
Study area	America	11	<0.001	3.10(1.80,5.36)	79.9	<0.001	0.777
	Europe	5	0.014	3.69(1.30,10.48)	56.1	0.059	
	Korea	1	0.007	3.34(1.38,8.08)	NA	NA	
Type of anticoagulant	Warfarin	11	<0.001	3.59(2.37,5.44)	16.9	0.283	0.213
	new oral anticoagulant(NOAC)	1	0.013	5.63(1.44,21.94)	NA	NA	
	Mixed	5	0.059	2.22(0.97,5.09)	89.9	<0.001	
Design of the control	Interrupt OAC Interrupted	10	<0.001	2.52(1.54,4.13)	76.8	<0.001	0.068
	Continue OAC Continued	4	0.085	4.33(0.82,22.92)	75.3	0.007	
	Non-OAC	1	0.007	7.84(1.74,35.35)	NA	NA	
	Mixed	2	<0.001	7.27(2.71,19.49)	0.0	0.592	
Patients’ number of subjects	Less than 2000	13	<0.001	3.64(2.22,5.96)	40.7	0.063	0.371
	More than 2000	4	0.028	2.51(1.11,5.68)	91.4	<0.001	
Study design	Randomized controlled trial(RCT)	5	0.040	2.42(1.04,5.65)	87.6	<0.001	0.298
	Non-RCT	12	<0.001	3.65(2.29,5.82)	43.3	0.055	
Type of heparin	Unfractionated heparin(UFH)	1	0.007	3.34(1.38,8.08)	NA	NA	0.856
	Low molecular weight heparin(LMWH)	5	<0.001	2.68(1.61,4.46)	0.0	0.655	
	Both of LMWH and UFH	11	<0.001	3.50(1.89,6.49)	82.6	<0.001	
Thromboemlism							
Year of study	Before2010	9	0.038	0.55(0.31,0.97)	2.7	0.412	0.198
	After 2010	8	0.513	1.41(0.50,3.99)	76.1	<0.001	
Type of disease	Atrial fibrillation(AF)	7	0.817	1.17(0.30,4.53)	86.8	<0.001	0.606
	Others	10	0.659	0.89(0.52,1.52)	0.0	0.876	
Study area	America	13	0.794	1.11(0.51,2.44)	79.9	<0.001	0.781
	Europe	3	0.090	0.32(0.09,1.19)	56.1	0.059	
	Korea	1	0.322	4.06(0.25,64.99)	NA	NA	
Type of anticoagulant	Warfarin	12	0.194	0.71(0.42,1.19)	5.1	0.395	0.550
	Mixed	5	0.893	1.11(0.25,4.94)	86.5	<0.001	
Design of the control	Interrupt OAC	11	0.656	1.21(0.52,2.82)	76.5	<0.001	0.355
	Continue OAC	3	0.198	0.42(0.11,1.58)	12.0	0.321	
	Non-OAC	2	0.963	0.93(0.04,22.99)	55.8	0.132	
	Mixed	1	0.449	0.33(0.02,5.93)	NA	NA	
Patients’ number of subjects	Less than 2000	12	0.317	0.75(0.44,1.31)	0.0	0.693	0.260
	More than 2000	5	0.519	1.64(0.36,7.43)	89.9	<0.001	
Study design	Randomized controlled trial(RCT)	5	0.371	1.83(0.49,2.00)	79.2	0.001	0.298
	Non-RCT	12	0.181	0.66(0.36,1.21)	26.5	0.184	
Type of heparin	Unfractionated heparin(UFH)	1	0.322	4.06(0.25,64.99)	NA	NA	0.856
	Low molecular weight heparin(LMWH)	5	0.159	0.60(0.29,1.22)	28.5	0.232	
	Both of LMWH and UFH	11	0.807	1.13(0.43,2.94)	70.6	<0.001	

### Publication bias

**Fig. 6:**
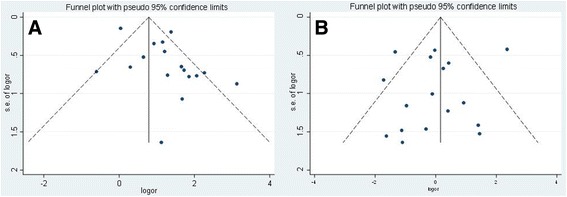
Funnel plots of major bleeding (**a**) and thromboembolism (**b**) for heparin-bridging and non-heparin bridging regiments

All the funnel charts were intuitively symmetrical and showed no obvious publication bias by the Egger test (P_majorbleeding_ = 0.039,P_thromboembolic events_ = 0.453) (Fig. 6).

## Discussion

From this meta-analysis of 25 studies and 35,944 patients, we found that among patients taking oral anticoagulants who underwent an elective operation or invasive procedure, heparin bridging increased the risk of major bleeding and overall bleeding events without reducing perioperative thromboembolism, all cause death, stroke or transient ischemic events compared with non-heparin bridging therapy.

The results of our analyses are consistent with several previously published studies. A meta-analysis including 34 studies and 12,278 patients receiving or not receiving bridging anticoagulation during interruption of VKA for elective procedures showed no significant difference in the risk of periprocedural arterial thromboembolism (OR 0.80; 95% CI, 0.42 to 1.54), rather HB is associated with a higher risk of major bleeding (OR 3.60; 95% CI, 1.52 to 8.50) [3]. On the other hand, a small number of studies were included in these comparative analyses between bridging and non-bridging therapy, and the summary estimates were mainly pooled from unadjusted effect estimates from observational studies. Similarly, it was discovered from the ORBIT-AF sub-trial that the risk of major bleeding events in patients receiving bridging therapy for atrial fibrillation was three to four times greater than that of patients who did not change anticoagulation regimens [7]. Moreover, bridging therapy did not reduce the risk of thrombosis in these patients. In a recent randomized controlled trial, 1884 patients with atrial fibrillation who had warfarin treatment interrupted for an elective operation or invasive procedure were randomized to either bridging anticoagulation therapy with low-molecular-weight heparin or matching placebo [8]. Bridging therapy was also associated with higher risk of major bleeding (3.2% vs. 1.3%, *P* = 0.005 for superiority) but similar risk of arterial thromboembolism (0.4% vs. 0.3%, *P* = 0.01 for noninferiority).

Our analyses are relevant to VKA-treated patients who require temporary interruption of oral anticoagulation for an elective operation or invasive procedure. It is critically important because more than 250,000 patients on long-term VKA undergo periprocedural assessment in North America each year alone [1] and this number is likely to increase. The perioperative management of patients receiving OACs is problematic because the medication must be discontinued to prevent excessive bleeding for many invasive and surgical procedures. Periprocedural bridging anticoagulation has been increasingly used in the past decade under the assumption that the higher risk of postoperative bleeding would be offset by the decreased risk of thromboembolism [9]. However, we found that patients undergoing heparin bridging, surprisingly, showed a probability of thromboembolic events which was quite similar with that of patients without any type of protective anticoagulation at the time of surgery. The findings of current analyses indicate that perioperative risk of thromboembolic events among patients requiring interruption of OAC treatment may have been overstated and may not be mitigated by bridging therapy. Indeed, the bridging anticoagulation may lead to increased risk of peri-procedure bleeding complications and should be used with great caution, especially in patients with low to moderate thromboembolic risk.

In both systematic review [9]and practice guidelines [1], the decision on “bridging” isleft to be individualized (based on clinical estimation of patients’ risks of thromboembolism and bleeding) and the type of operation by balancing expected benefits and harms. A systematic review identifying thirty-one reports suggested that most patients undergoing dental procedures, joint and soft tissue injections and arthrocentesis, cataract surgery, and upper endoscopy or colonoscopy with or without biopsy can undergo the procedure without alteration of their OAC regimen. But for others, the decision whether to bridge with intravenous heparin or subcutaneous LMWH should be individualized, reliance on copious clinical experience and factoring in outcomes of patients hematological studies and preference. [9]. The 2012 guidelines on the optimal management of patients receiving OACs during the perioperative period recommends perioperative antithrombotic management should be based on risk assessment for thromboembolic and hemorrhagic risks. In patients with mechanical heart valve (s), atrial fibrillation, or VTE at higher risk for thromboembolism, they suggest bridging anticoagulation instead of no bridging during VKA interruption (Grade 2 with level of evidence C); no bridging instead of bridgingfor patients at low riskfor thromboembolic events (Grade 2C) [1]. In current analysis, we included studies on different procedures and patients with varying degrees of thromboembolic and bleeding risk profiles. Although pre-defined subgroup analyses and meta-regressions were performed to explore the between-group heterogeneity, these analyses were only able to assess the study-level values provided by the included publications rather than individual patients. We did not have the capability to group the studies according to the type of surgery or patients’ risk profiles. Therefore, our finding does not preclude the possibility that bridging anticoagulation yields more benefit than harm in certain group of patients with low bleeding risk receiving procedures with high thromboembolic risk.

The main strength of our study is that it incorporated the most comprehensive studies (19 observational studies and 6 randomized controlled trials) since 2005 and included not only warfarin but also new oral anticoagulants. There are several limitations to this analysis. First, most of included studies (19 of 25 studies) were observational studies. We acknowledge that the control groups of the observational studies might consist of low thromboembolic-risk patients compared with bridged groups. Therefore, there is the possibility of treatment and control groups having different thromboembolic risks at baseline, which could lead to a risk of systemic bias in regard to which patients were bridged or not. This could partially explain the similar thromboembolic risk between bridged and non-bridged patients in this meta-analysis. It is possible that bridging therapy may have reduced a high thromboembolic risk in these high-risk, bridged patients to the level similar with that in the lower risk, non-bridged patients. Even though, we did not find significant heterogeneity between summary estimates from observational studies and RCTs, this bias has little chance of changing the overall conclusions of this analysis. Second, the control group consisted of both interruption of oral anticoagulants and continued oral anticoagulation. The decision on which treatment method to apply is based on the type of surgery and the risk of bleeding versus thrombosis of original studies. Therefore, in the control group, there may be differences in the risk of bleeding and thrombosis between interruption and continuation of oral anticoagulants. Finally, bleeding risks may vary between major and minor surgeries. However, most of the individual studies didn’t report bleeding risk according to the type of procedure, which made us unable to perform analyses to account for such potential variation.

## Conclusions

In summary, the results of this meta-analysis suggest that compared to non-bridged patients, OAC-treated patients receiving periprocedural heparin-bridging therapy seem to be at increased risk of bleeding and at similar risk of thromboembolic events and all cause death. The 2012 antithrombotic practice guidelines of American College of Chest Physicians (ACCP) recommended heparin bridging therapy should be undertaken into consideration in light with the pros and cons balanced by individual patient’s thromboembolic risk and procedural bleeding risk. This study has some level of methodological limitations, therefore, more high-quality large-scale clinical randomized controlled trials are still needed to better guide decision making on this clinical practice.

## Abbreviations

CAD: Coronary artery disease
HB: Heparin bridging
MI: Myocardial infarction
NHB: Non-heparin bridging
NOAC: New oral anticoagulant
OAC: Oral anticoagulant
TIA: Transient Ischemic Attack
VKA: Vitamin K antagonist
